# Early responses of *EGFR* circulating tumor DNA to EGFR tyrosine kinase inhibitors in lung cancer treatment

**DOI:** 10.18632/oncotarget.12373

**Published:** 2016-09-30

**Authors:** Fumio Imamura, Junji Uchida, Yoji Kukita, Toru Kumagai, Kazumi Nishino, Takako Inoue, Madoka Kimura, Kikuya Kato

**Affiliations:** ^1^ Department of Thoracic Oncology, Osaka Medical Center for Cancer and Cardiovascular Diseases, Osaka, Japan; ^2^ Research Institute, Osaka Medical Center for Cancer and Cardiovascular Diseases, Osaka, Japan

**Keywords:** lung cancer, EGFR, EGFR-TKI, mutation, response evaluation

## Abstract

**Objectives:**

Early evaluation of the effect of treatment is helpful in the management of cancer patients. Circulating biomarkers are an ideal tool for this if they are highly specific to tumors and respond rapidly to tumor volume changes. Circulating tumor DNA (ctDNA) is one such candidate. We conducted a prospective study to test the utility of *EGFR* ctDNA in early evaluation of EGFR-TKI effects.

**Results:**

Twenty-one patients with EGFR-mutant lung cancer who were naïve to EGFR-TKI were enrolled. PM scores of *EGFR* ctDNA with activating mutations decreased rapidly in response to EGFR-TKI. Of the 14 patients with positive pretreatment PM scores, complete disappearance of major EGFR ctDNA was observed in 14.3%, 42.9%, and 57.1% on days 2 – 4, 8, and 15, respectively. These responses of *EGFR* ctDNA were most prominent among the measures used to evaluate responses, and correlated with early radiologic responses evaluated by chest X-rays.

**Materials and methods:**

*EGFR* ctDNA in serial plasma samples was amplified and 10^5^ copies were sequenced with a next-generation sequencer. Plasma mutation (PM) score was defined as the number of reads containing deletions/substitutions in 10^5^ *EGFR* cell free DNA (cfDNA). When *EGFR* mutation in ctDNA was the same as that detected in cancer tissue, the ctDNA was defined as major *EGFR* ctDNA.

**Conclusions:**

The results indicate the usefulness of ctDNA as a highly specific biomarker for prediction of early response to treatment and that it can be applied to various types of cancer.

## INTRODUCTION

Cancer is one of the leading causes of death in advanced countries. Surgery, radiation, and chemotherapy are mainstays of cancer treatment. Recently, molecular-targeted therapy has attained great success, and immunotherapy through immune checkpoint blockade is being rapidly developed for certain types of cancer. In an era with multiple treatment choices for cancer, evaluating the effect of each treatment, especially in the early stage, is undoubtedly critical to patient care. Although computed tomography (CT) is a standard tool for response evaluation in almost all solid cancers, it usually takes weeks for radiologic responses to develop. Positron emission tomography (PET), which reflects the metabolic activity of tumors, is reported to enable early evaluation of treatment, but repetition of PET is not realistic [1, 2]. Thus, effective and repeatable methods that reflect tumor volume changes are required.

Circulating biomarkers are useful for assessing tumor burden, and therefore patient response during treatment. Circulating tumor DNA (ctDNA) which carries cancer-specific genetic changes is detected in the plasma of cancer patients and being increasingly employed for various purposes [3, 4]. ctDNA carrying cancer-specific changes offers the advantage of being highly specific. Recently, the utility of ctDNA in monitoring tumor burden, minimal residual disease, and acquired resistance has received extensive research attention [5]. Since cancer cells undergo multiple genetic changes, ctDNA carrying these molecular changes will gain more importance in the future.

*EGFR* ctDNA with activating mutations is one of the representative types of ctDNA detected in the plasma of patients with NSCLC harboring these mutations. EGFR tyrosine kinase inhibitors (EGFR-TKIs) show dramatic effects on NSCLC with activating *EGFR* mutations [6, 7]. The responses of *EGFR* ctDNA to EGFR-TKI treatments provide an ideal model to investigate the role of ctDNA in monitoring cancer treatment. Our preceding study showed that *EGFR* ctDNA levels reflected the effect of EGFR-TKI in *EGFR*-mutant lung cancer, i.e. the amount of *EGFR* ctDNA decreased in response to EGFR-TKI treatment reflecting the radiologic responses at least in part [8, 9]. We hypothesized that monitoring ctDNA will assist early prediction for treatment responses. To analyze the early response of *EGFR* ctDNA to EGFR-TKI treatments, we conducted a prospective study, wherein the quantitative changes in *EGFR* ctDNA were evaluated in the first 3 weeks of EGFR-TKI treatment for lung cancer with activating *EGFR* mutations.

## RESULTS

### Detection of *EGFR* ctDNA

Twenty-one NSCLC patients were enrolled into this study between August 2013 and April 2014. The patient characteristics are shown in Table 1. PM scores were obtained before beginning the EGFR-TKI treatment, and in periods 1, 2, and 3 for all patients. The pretreatment PM score of the major *EGFR* ctDNA was positive in 14 patients (66.6%) and negative in 7 patients (33.3%). This positive rate was in agreement with that observed in our preceding study [13]. Among 7 patients with negative pretreatment PM scores, a transient peak of major *EGFR* ctDNA appeared in 2 patients during period 1, but no major *EGFR* ctDNA was detected throughout period 1 to 3 in the remaining 5 patients.

**Table 1:** Patient characteristics

Age	mean (range)	68 (53 – 87)
Sex	men/women	6/15
Stage	M1a/M1b	4/17
PS	0/1/2	3/14/4
EGFR mutation type (cancer tissue)	Exon 19 deletion/L858R	10/11
Histology	Ad/AdSq	20/1
EGFR-TKI	Gefitinib/Erlotinib	14/7
Preceding treatment		
	Surgery	4
	Chemoradiation	4
	Chemotherapy	1
	Palliative radiation	3
	None	9
Best response to EGFR-TKI		
	CR/PR/SD/NE	1/17/1/2

### Rapid decrease in *EGFR* ctDNA in response to EGFR-TKIs

### Major *EGFR* ctDNA

**Figure 1:**
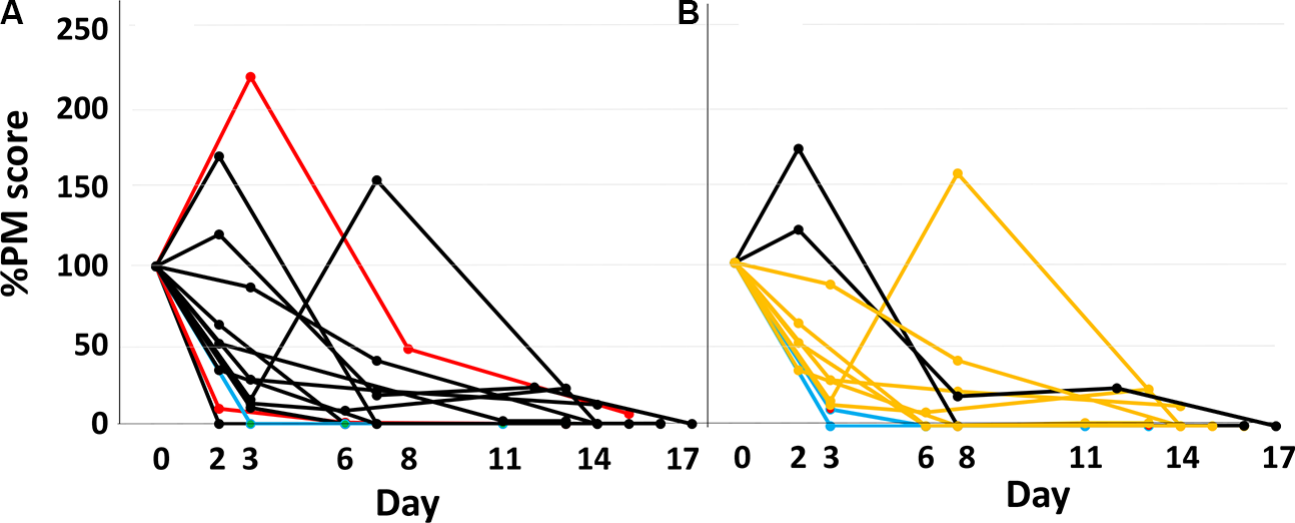
Response pattern of major *EGFR* ctDNA to EGFR-TKI treatment during the first 14 days (**A**) All patients with positive pretreatment *EGFR* ctDNA values. Red, black, and blue lines represent CR, PR, and SD cases, respectively. (**B**) Patients in whom the response to an EGFR-TKI during the first 14 days was evaluable in chest X-p. Black, orange, and blue lines represent PR, MR, and SD cases, respectively. CR, complete regression; PR, partial regression; MR, minor regression; SD, stable disease.

**Figure 2:**
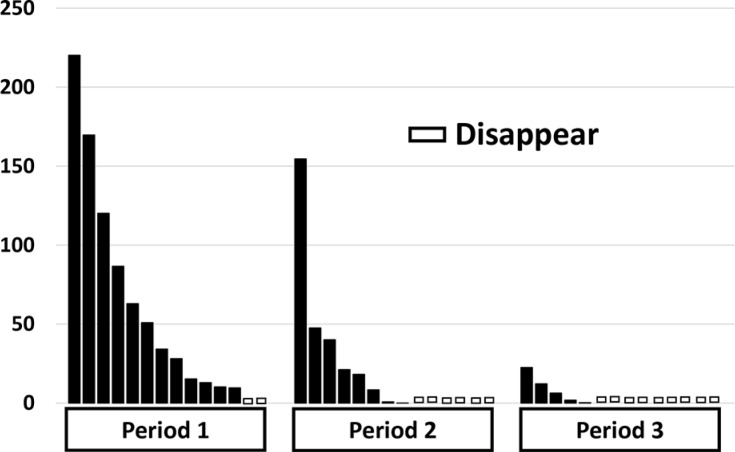
Percent values of *EGFR* ctDNA in 14 patients in the periods 1 – 3 in comparison with the pretreatment values

The percent change in PM scores of major *EGFR* ctDNA in patients with positive pretreatment PM scores are shown in Figure 1A. PM scores showed a monotonous rapid decrease in the majority of patients, whereas a transient peak was observed in 4 patients. The percent change in PM scores for each period is shown for all 14 patients with positive pretreatment PM scores (Figure 2). Complete disappearance of the major *EGFR* ctDNA was observed in 14.3%, 42.9%, and 57.1% of the patients in periods 1, 2, and 3, respectively. Percent PM scores of the major *EGFR* ctDNA decreased to less than 10% in 21.4%, 64.3%, and 84.6% of the patients in periods 1, 2, and 3, respectively. The transient peaks of the major *EGFR* ctDNA also disappeared in period 3 in 2 patients with negative pretreatment PM scores (data not shown).

### Minor *EGFR* ctDNA

**Figure 3:**
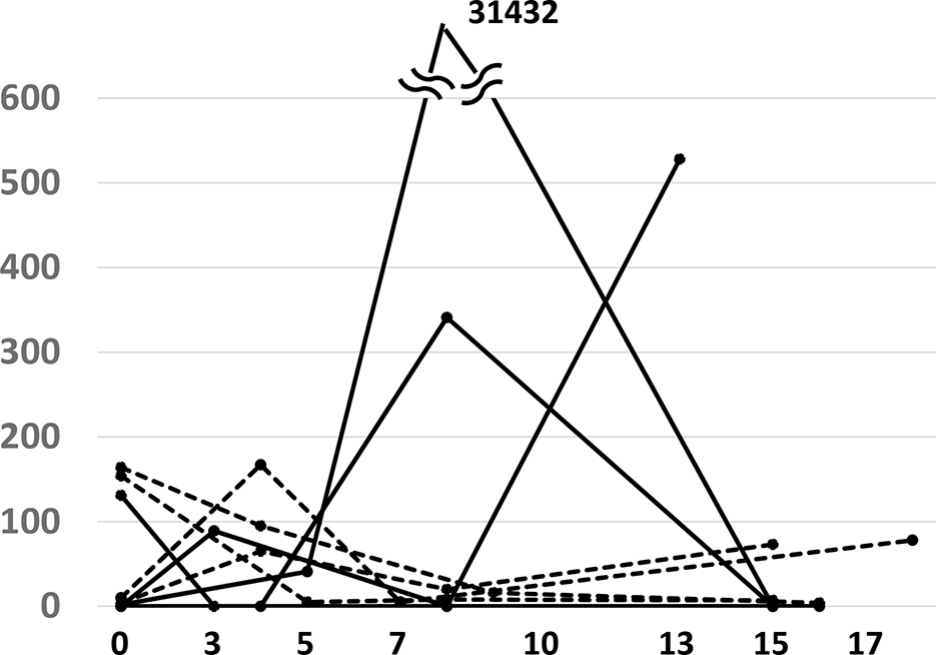
Response of minor *EGFR* ctDNA to EGFR-TKI treatment during the first 14 days Minor ctDNA appeared in 8 patients. Solid and dashed lines represent *EGFR* ctDNA with activating mutations (4 cases) and T790M (4 cases), respectively.

Minor *EGFR* ctDNA was detected in 8 patients (38.1%): activating mutations in 4 patients and T790M in 4 patients. In 3 patients, only minor *EGFR* ctDNA was detected with no major *EGFR* ctDNA: activating mutation in 1 patient and T790M in 2 patients. The early response patterns of minor *EGFR* ctDNA are shown in Figure 3. The minor *EGFR* ctDNA disappeared in 5 patients, but persisted during the observation period in 3 patients (1 patient with an activating *EGFR* mutation and 2 patients with T790M). All these minor ctDNA disappeared in the subsequent evaluation (data not shown).

We have tested the correlation between patient characteristics and detection of minor EGFR ctDNA (Table 2). The presence of minor EGFR cDNA showed some correlation to stage (M1a or M1b), mutation type, and the presence or absence of previous treatments, although statistically not significant probably due to small number of the patients. The results may suggest increased clonal divergence of cancer cells accompanied by disease progression.

**Table 2:** Correlation between ctDNA patterns and patient characteristics

Category	Metastasis^1^		EGFR^2^		Prior treatment^3^	
	M1a	M1b	L858R	Ex19del	No	Yes
Major only	3	8	7	4	7	4
Minor ± Major	1	7	4	4	2	6
No response	0	2	2	0	0	2
Minor (%)	25.0	41.2	30.8	50.0	22.2	50.0
Total	4	17	13	8	9	12

1 *p* = 0.199,

2 *p* = 0.223,

3 *p* = 0.199.

### Radiologic response and major *EGFR* ctDNA

CT examination to evaluate responses was planned to perform on between the 29th and 43th day of the treatment. In the actual study, the first CT to evaluate responses was performed on the 39th day as a median. Radiologic response was evaluated by CT in all patients. CR, PR, SD, and NE were attained in 1, 17, 1, and 2 patients, respectively; none of the patients experienced PD. The response rate was 85.7%. The decrease in percent PM scores was not translated simply to better radiologic response by CT (Figure 1A). In lung cancer, treatment response in chest lesions can be evaluated by chest X-p examination at a stage earlier than CT, although 8 patients (38.1%) had no evaluable targets in chest X-p examinations in this study. Chest X-p examinations performed during the first 1 week showed PR, MR, SD in 2, 9, and 2 patients out of 13 patients with evaluable targets in chest X-p, respectively; no PD was observed. Interestingly, responses evaluated by chest X-p examinations appeared to be correlated with ctDNA responses (Figure 1B).

### Application of a mathematical model to ctDNA

**Figure 4:**
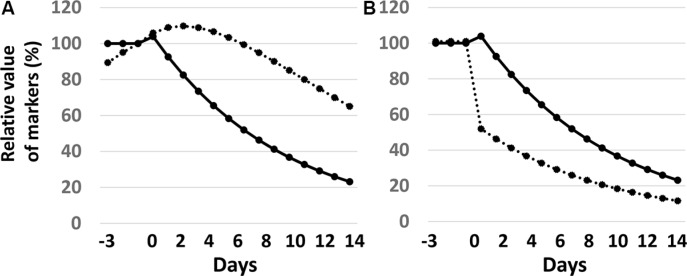
Mathematical models of circulating biomarkers The half-life of tumor regression is postulated to be 6 days. (**A**) Effect of half-life on concentration of biomarkers. Solid and dashed lines represent biomarkers with a half-life of 2 hours and 7 days, respectively. (**B**) The effect of sensitive cancer cell volume on concentration of biomarkers. Solid and dashed lines represent biomarkers in the sensitive volume of cancer cells to be 100% and 50%, respectively. The half-life of the biomarker is postulated to be 2 hours.

The application of an exponential decrease model in cytotoxicity by EGFR-TKI treatments appeared to fit for EGFR-TKI-induced tumor cell reduction (Supplementary Figure S2). According to this model, the half-life of a circulating biomarker has remarkable effects on its blood concentration. The half-life of ctDNA is reported to be approximately 2 hours, which is distinct from the 3 – 11 days of the half-life of CEA. The changes in circulating ctDNA and CEA values would be very different even under the same condition of tumor destruction (Figure 4A). The change in the blood concentration of a biomarker with a short half-life reflects the real-time volume of destructed cancer cells compared to that with a long half-life.

The mathematical model indicates that the area under the curve (AUC) of a %-expressed concentration curve of a biomarker represents the volume of destructed cancer cells, and the total AUC value depends on the half-life of the biomarker and the volume of sensitive tumor cells, but not on the half-life of tumor destruction (Supplementary Figure S1). When a treatment is effective on a limited tumor volume, the percent PM score decreases rapidly (Figure 4B). In Figure 1B, 3 types of PM curves are observed: one is that with an early peak (ex. black curves). The steep slope and the large AUC of these curves indicate a tumor shrinkage of appreciable volume occurring with a short half-life. The second type has a looser slope (mostly orange curves), indicating a longer half-life of tumor shrinkage. The third type has a steep slope along with a small AUC. This pattern is fit for the destruction of a relatively small volume of tumor cells with a short half-life.

### Biomarkers and *EGFR* ctDNA

**Figure 5:**
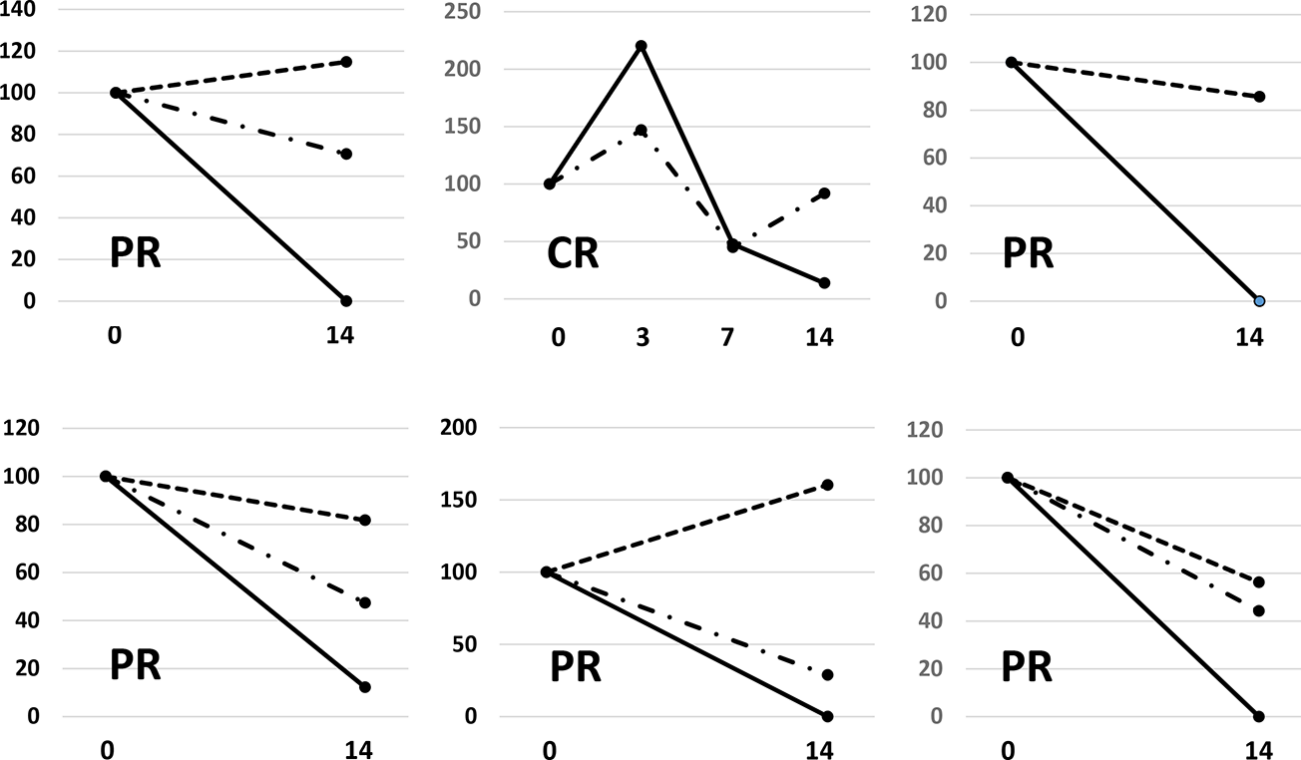
Changes in various circulating biomarkers during the first two weeks in 6 patients with positive pretreatment values both in *EGFR* ctDNA and at least one widely used tumor marker Continuous lines, dashed lines, and long dashed dotted lines represent *EGFR* ctDNA, CEA, and CYFRA, respectively. Figure 1.

CEA, CYFRA, and NSE were tested in 21, 16, and 13 patients before treatment, and were found to be positive in 13 (61.9%), 7 (43.8%), and 1 (7.7%) patients, respectively. The pretreatment value of at least one out of three biomarkers was elevated in 19 patients (90.5%). Among these, data sets were available for 6 patients for comparing CEA or CYFRA values with PM scores in the first 14 days of EGFR-TKI treatment. Percent changes in biomarkers and *EGFR* ctDNA in these 6 patients are shown in Figure 5. The results show that *EGFR* ctDNA was more sensitive to the treatment than the widely used biomarkers, at least in the early phase of treatment. *EGFR* ctDNA showed a strong tendency to disappear in patients with good radiologic responses.

## DISCUSSION

To our knowledge, this is the first report of a prospective analysis to clarify early *EGFR* ctDNA responses during therapy with EGFR-TKIs. These results provide strong evidence supporting the usefulness of ctDNA harboring tumor-specific changes in the early evaluation of treatment responses. The plasma concentration of *EGFR* ctDNA responded rapidly to EGFR-TKI treatments. Between days 2 and 4, PM scores decreased to less than 10% of the pretreatment value in 21.4% of the patients and even reached 0 in 14.3% of the patients. The response of *EGFR* ctDNA was most rapid among all the circulating biomarkers tested in this study. Despite the presence of exceptions such as prostate specific antigen (PSA), biomarkers generally lack high specificity. Most typically, the serum value of CEA is known to increase in smokers. *EGFR* activating mutations are known to be highly specific to lung cancer, and therefore the ctDNA harboring these mutations is also highly specific to lung cancer. Although most of these cancer-related mutations may be specific to cancer but not to its specific type, a high specificity to cancer per se is an outstanding advantage of ctDNA as a biomarker. It is also advantageous that basically the same method is applicable to quantify all types of ctDNA.

However, the rapid reaction of *EGFR* ctDNA was not translated simply to the radiologic responses evaluated by CT. Our former study had also shown that the correlation between *EGFR* ctDNA responses and radiologic responses by CT was not simple [9]. On the contrary, early changes in *EGFR* ctDNA appeared to correlate well with the early radiologic responses evaluated by chest X-p, even though ctDNA responses were more evident than those observed in chest X-p. To understand this discrepancy, we constructed a mathematical model of circulating biomarkers. The model showed that ctDNA, which is characterized by a short half-life, could reflect the volume of destructed cancer cells in almost a real-time fashion. The early change in PM score can be considered to reflect early tumor responses to EGFR-TKI treatments, and hence should show good correlation to changes in chest X-p but not in CT. Early decrease in *EGFR* ctDNA indicates the presence of cancer cell populations that are sensitive to EGFR-TKIs. On the contrary, the entire tumor is a mixture of heterogeneous cancer cell clones with varying sensitivity to EGFR-TKIs. In addition, release of ctDNA by destruction of cancer cells would depend on the location and size of the tumors, and access to blood vessels. Since response evaluation by CT, which evaluates distal effects, can be compromised by these factors, it showed no simple correlation with early changes in *EGFR* ctDNA. It is important to note that our previous study showed that the ratio of minimum PM score during the first 6 months of treatment to the pre-treatment score, Lmin0-6 index, could be a good predictor of radiologic responses by CT [9].

Early response of EGFR ctDNA to EGFR-TKI treatment was already showed by Marchetti A, et al. [10]. However, they concluded that the amount of ctDNA depended mainly on the amount of tumor cells released in the bloodstream that could decrease under treatment, and necrotic or apoptotic cells could only be marginally involved in the process. This conclusion is fairly different from ours. In EURTAC trial, EGFR mutations in circulating free DNA (cfDNA) was measured by the peptide nucleic acid–mediated 5′ nuclease real-time polymerase chain reaction (TaqMan) assay, and suggested a possibility of a novel surrogate prognostic marker of the L858R mutation in cfDNA [11].

In this analysis, we adopted a liberal approach, setting the threshold of detection as LOD. Liberal statistical approaches may be useful for extraction of data, but might be affected by false positives. The possibility of false positives should be considered, especially for exon 19 deletions as minor mutations, because the specificity for Ex19del is 73% with LOD. Taken together, the results presented in this manuscript indicate the usefulness of ctDNA as a highly specific biomarker, which contributes to the prediction of an early response to treatments and can be applicable for various types of cancer. ctDNA is also critical in evaluating the size of responses when suitable indices such as Lmin0-6 are applied. The combination of image analysis and ctDNA monitoring may provide a better way to evaluate the responses to cancer treatment.

## MATERIALS AND METHODS

### Patient accrual and sample collection

We performed a prospective, single-center study to test the role of *EGFR* ctDNA for the early evaluation of treatment response in patients with *EGFR*-mutant NSCLC. This study was approved by the local institutional review committee. Eligibility criteria included patients with adenocarcinoma carrying activating *EGFR* mutations, and naïve to EGFR-TKI, who provided written informed consent. *EGFR* mutations in cancer tissue were detected by PNA-LNA PCR clump method. During EGFR-TKI treatments, blood samples (6 mL each) were serially collected before treatment, on days 2 – 4 (period 1), 8 (period 2) and 15 (period 3) with an allowance of ± 2 days.

### Radiologic evaluation of responses

Tumor response to treatment was determined by computed tomography (CT evaluation) and was reviewed according to RECIST (response evaluation criteria in solid tumors) guideline [10]. Responses were classified as complete response (CR), partial response (PR), stable disease (SD), or progressive disease (PD). CT examination to evaluate responses was planned to perform on between the 29th and 43th day of the treatment. For early radiologic evaluation, chest X-ray evaluation was performed at least twice in 3 weeks for all patients (chest X-p evaluation). Due to evaluation in the early stage, response was categorized as CR, PR, MR (minor response), SD, and PD in chest X-p evaluation. The data cut-off time for response evaluation was 4 January 2016.

### Detection of *EGFR* mutations and PM score

Exons 19, 20, and 21 of the *EGFR* gene in patient plasma DNA were independently amplified by the polymerase chain reaction, and deep sequencing was performed with an Ion Torrent PGM [11, 12]. More than 10^5^ reads were obtained for each exon region. Since each read was for a single molecule, we were able to estimate the relative ratio of mutational alleles to wild type *EGFR* cell-free DNA (cfDNA). The plasma mutation (PM) score was defined as the number of reads containing deletions/substitutions per 10^5^ *EGFR* cfDNA reads. Output data from the Ion Torrent PGM were processed as previously described [13, 14].

From the basic analysis with our system, we set 2 thresholds for PM score: a limit of detection (LOD) and a limit of quantitation (LOQ) [14]. In the present study, PM score was considered as positive when it exceeded LOD. The LOD was 7, both for the exon 19 deletion and L858R. The LOD for T790M was 60. To evaluate the sensitivity and specificity of this system in the clinical setting, we completed a multi-institutional prospective study, where *EGFR* cDNA levels at initial diagnosis were evaluated in 288 patients with lung adenocarcinoma [15]. The sensitivity for detecting activating *EGFR* mutations was found to be approximately 70% in advanced NSCLC. The specificities with LOD are 74% for Ex19del, 94 % for L858R, and 94% for T790M. Percent PM score was defined as the ratio (%) of PM score against pretreatment value. When the *EGFR* mutation in ctDNA was the same as that detected in cancer tissue, the ctDNA was defined as major *EGFR* ctDNA. The *EGFR* ctDNA differing from major *EGFR* ctDNA or one carrying T790M was defined as minor *EGFR* ctDNA.

### Circulating biomarkers

*EGFR* ctDNA harboring cancer-specific changes is considered to be a biomarker that is highly specific to lung cancer. We compared it with the widely used biomarkers for lung cancer. We selected carcinoembryonic antigen (CEA), cytokeratin-19 fragment (CYFRA), and neuron-specific enolase (NSE). When the pretreatment values of these markers exceeded their cut-off values, they were serially measured and used to evaluate responses.

### Mathematical models

A mathematical model was constructed to analyze the changes during EGFR-TKI treatments in plasma concentrations of biomarkers including *EGFR* ctDNA (Supplementary Figure S1). Because of intensive cytotoxicity of EGFR-TKIs, we hypothesized exponential decrease of cancer cell number just after the initiation of EGFR-TKI treatment, until when it is almost constant during a short time period (10 days). The half-lives of *EGFR* ctDNA and CEA were set as 2 hours and 7 days, respectively, as previously reported [16, 17]. It was also postulated that each cancer cell contained one biomarker molecule which is released into blood only upon cancer cell destruction. These assumptions do not compromise the generalizability of the results. To confirm exponential reduction of tumor cell number in the early phase of EGFR-TKI treatment, patients who had 3 or more points of positive PM scores during the first 3 weeks of the treatment were selected from all the participants in either of our *EGFR* ctDNA studies. Their PM scores during the first 3 weeks of the treatment showed that logarithm of PM scores had almost linear correlations with time. Because of a short half-life of ctDNA, this means that EGFR-TKI-induced reduction of tumor cell number can be considered approximately exponential during the first 3 weeks (Supplementary Figure S2).

## SUPPLEMENTARY MATERIALS FIGURES


