# Supplementary material for: Early responses of EGFR circulating tumor DNA to EGFR tyrosine kinase inhibitors in lung cancer treatment
Source: Oncotarget. 2016 Sep 30;7(44):71782–9. doi: 10.18632/oncotarget.12373 (PMC5342122; doi:10.18632/oncotarget.12373)
Supplement: Supplementary file 1 [file oncotarget-07-71782-s001.pdf]

Early responses of *EGFR* circulating tumor DNA to EGFR tyrosine kinase inhibitors in lung cancer treatment

Supplementary Materials

The number of cancer cells, with N(0) at time 0, is postulated to decrease exponentially with a half-life of L from time 0, when the treatment starts. Each cancer cell is postulated to contain one molecule of each biomarker, and the biomarker molecules are released into blood only upon cancer cell destruction. The number of biomarker molecules released during time (T, T+dT) ($T \geq 0$) is:

$$N(0)*(\frac{\log 2}{L})*\exp(-\log 2*T/L)*dT$$

Due to the half-life H of the biomarker, the number of biomarker molecules released in this time period decreases at time t ($t \geq T$) to:

$$n(T) = N(0)*(\frac{\log 2}{L})*\exp(-\log 2*T/L)*dT*\exp[-\log 2*(t-T)/H]$$

The number of biomarker molecules at time t, N(t), can be calculated by integrating n(T) from time 0 to t. As a result:

$$N(t) = \int_0^t N(0)*(\frac{\log 2}{L})*\exp(-\log 2*T/L)*\exp[-\log 2*(t-T)/H]dT$$

$$= N(0)*(\frac{H}{L-H})*[\exp(-\log 2*t/L) - \exp(-\log 2*t/H)]$$

For simplicity, it is postulated that 10% of cancer cells are destroyed every day but the total cell number is stable in the short period before starting the EGFR-TKI treatment. The number of cancer cells before and after the beginning of EGFR-TKI treatment is described with H=0.083 days (2 hours) as a model of ctDNA and H=7 days as a model of CEA, respectively. The value of L is plotted as 4, 6, and 8 days.

Set K ($0 \leq K \leq 1$). If 100K% of the total tumor cells are sensitive to an EGFR-TKI and are destroyed exponentially by the treatment, the number of biomarker molecules released into the blood at time t is K*N(t). The total number of released molecules, T is:

$$T = \int_0^\infty K * N(t) dt$$

$$= K*(\frac{H}{L-H})*N(0)*\int_0^\infty [\exp(-\log 2*t/L) - \exp(-\log 2*t/H)]dt$$

$$= K*(\frac{H}{L-H})*N(0)*(\frac{L}{\log 2} - \frac{H}{\log 2})$$

$$= \frac{1}{\log 2}*K*H*N(0)$$

When the number of tumor cells is expressed as the ratio to N(0) (the same as N(0)=1), the AUC of the biomarker curve depends on H and K, but is independent of L. With a longer L, the biomarker curve shifts to the right with the same AUC. With K (<1), the biomarker curve shifts to the left with a smaller AUC.

Figure A. Simulated curves of the blood concentration of a biomarker with H=0.056 for L of 4, 6, and 8.

Because L≫H,

$$\exp(-\log2^{*}t/L) \gg \exp(-\log2^{*}t/H)$$

Reflecting the short half-life of the biomarker, the shape of the curves after day 0 traces the curve of cancer regression and also volume of destructed cancer cells.

Figure B. Simulated curves of the blood concentration of a biomarker with H=7 for L of 4, 6, and 8. Supplementary Figure1

We selected all of the patients who had 3 or more positive PM scores during the first 3 weeks in EGFR-TKI treatment from the participants in any of our *EGFR* ctDNA researches. Eight patients were selected, and time courses of *EGFR* ctDNA values in these patients are shown. Horizontal and vertical axis represent time (day) after beginning EGFR-TKI treatment and *EGFR* ctDNA values in natural logarithmic scale, respectively. The results show that these 2 indices have almost linear correlations, indicating exponential reduction in *EGFR* ctDNA in the early EGFR-TKI-induced tumor shrinkage. As shown in Supplementary figure 1, the amount of *EGFR* ctDNA at time t, N(t), is:

$$N(t) = N(0)^{*}\left(\frac{H}{L-H}\right)^{*}[\exp(-\log2^{*}t/L) - \exp(-\log2^{*}t/H)]$$

In L>>H,

$$N(t) \fallingdotseq N(0)^{*}\left(\frac{H}{L-H}\right)^{*}\exp(-\log2^{*}t/L)$$

This means that our hypothesis in Supplementary figure 1 that reduction of tumor cell number is exponential (log kill) is appropriate in the early EGFR-TKI-induced tumor shrinkage.

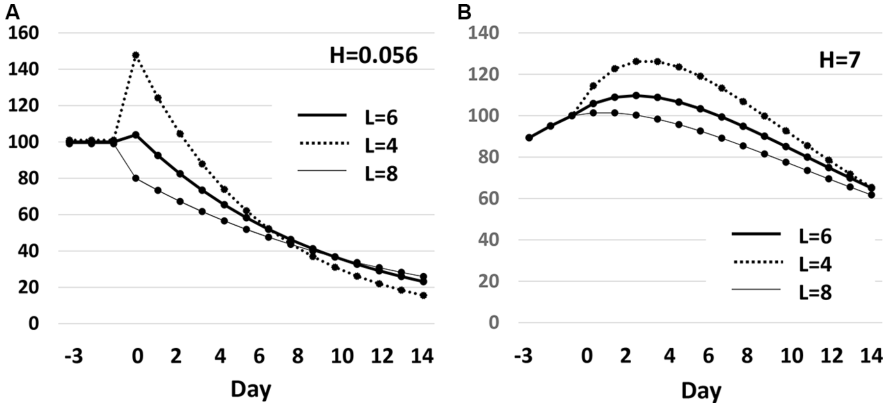

Supplementary Figure S1: (**A**) Simulated curves of the blood concentration of a biomarker with H=0.056 for L of 4, 6, and 8. (**B**) Simulated curves of the blood concentration of a biomarker with H=7 for L of 4, 6, and 8.

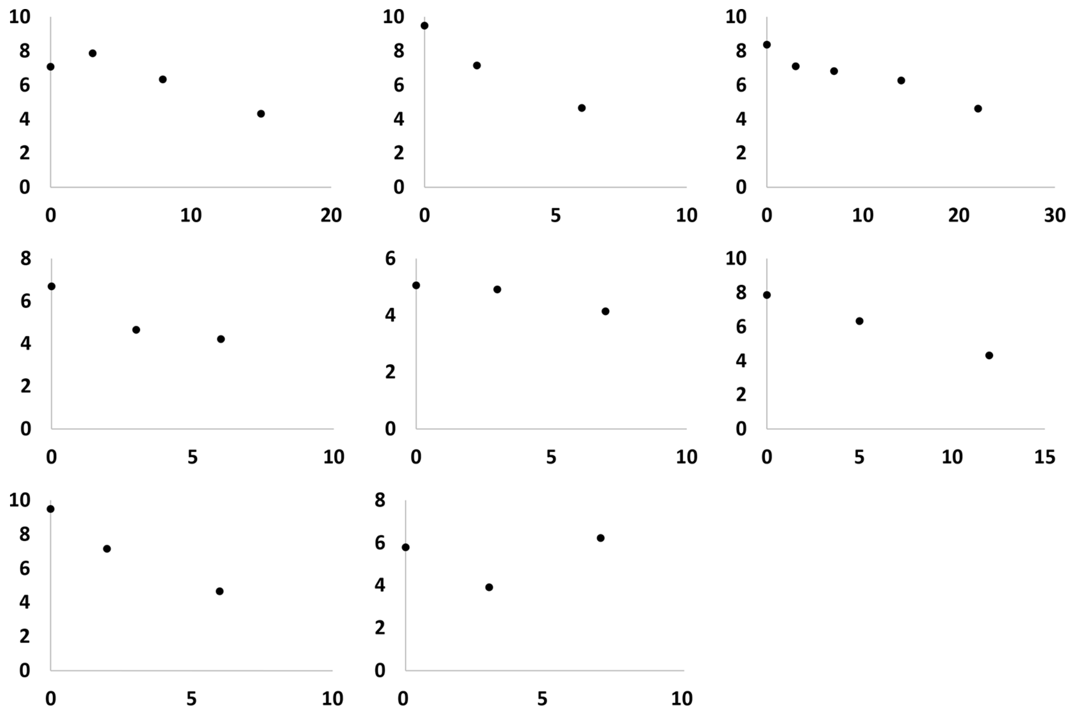

Supplementary Figure S2: Eight patients were selected, and time courses of EGFR ctDNA values in these patients are shown. Horizontal and vertical axis represent time (day) after beginning EGFR-TKI treatment and EGFR ctDNA values in natural logarithmic scale, respectively.
